# Effectiveness of Controlled Ovarian Stimulation for Oocyte Preservation in Oncologic Patients: Insights from DuoStim Protocol

**DOI:** 10.3390/jcm14228062

**Published:** 2025-11-14

**Authors:** Andrea Panattoni, Maria Magdalena Montt Guevara, Ilaria Marzi, Koray Görkem Saçıntı, Francesca Papini, Chiara Maggiorano, Sara Macaluso, Elena Casarosa, Tommaso Simoncini, Paolo Giovanni Artini, Vito Cela

**Affiliations:** 1 Division of Assisted Reproductive Medicine and Infertility, Obstetrics and Gynecology Unit, Department of Maternal and Child Health, Pisa University Hospital (AOUP), 56126 Pisa, Italy; andreapanat@gmail.com (A.P.); ilariamarzi1@gmail.com (I.M.); papinifra@libero.it (F.P.); e.casarosa@ao-pisa.toscana.it (E.C.); pgartini@gmail.com (P.G.A.); 2 Division of Obstetrics and Gynecology, Department of Clinical and Experimental Medicine, University of Pisa, 56126 Pisa, Italy; maria.montt@unipi.it (M.M.M.G.); chiara.maggiorano@gmail.com (C.M.); saramacaluso95@gmail.com (S.M.); tommaso.simoncini@unipi.it (T.S.); 3 Department of Obstetrics, Gynecology and Reproductive Sciences, Yale School of Medicine, New Haven, CT 06510, USA; mail@koraygorkemsacinti.com; 4 Division of Epidemiology, Department of Public Health, Faculty of Medicine, Hacettepe University, 06800 Ankara, Turkey

**Keywords:** oncofertility, DuoStim, controlled ovarian stimulation (COS), oocytes retrieval, Follicular Output Rate (FORT), Follicle Oocyte Index (FOI), Ovarian Sensitivity Index (OSI)

## Abstract

**Background/Objectives**: Fertility preservation is a key component of oncological care. This study evaluated the effectiveness of different controlled ovarian stimulation (COS) protocols, including dual stimulation (DuoStim), for oocyte preservation, with a specific focus on breast cancer patients, and aimed to identify predictors of mature oocyte yield. **Methods**: A retrospective single-center study was conducted on 203 women under 40 years undergoing fertility preservation before cancer treatment between August 2013 and May 2024 at the Fertility Unit of the University Hospital of Pisa. COS protocols were stratified by menstrual cycle phase: early follicular (EFP), late follicular (LFP), luteal (LP), and DuoStim. The primary outcome was fertility preservation, assessed by the number of mature oocytes retrieved (MII). Independent predictors of oocyte yield were assessed using multivariable Poisson regression. **Results**: A total of 244 COS cycles were analyzed. The DuoStim group showed a lower median number of MII oocytes collected during the second stimulation compared to EFP, LFP, and LP (all adjusted *p*-value < 0.05, FDR); however, cumulative MII counts across both stimulations were comparable to other protocols. Oocyte maturity rates were similar across groups. Multivariable analysis identified AMH and AFC, but not age, basal FSH, hormonal parameters, and year of cryopreservation, as independent predictors of MII oocyte yield. **Conclusions**: COS is effective for fertility preservation across different cycle phases without delaying cancer treatment. DuoStim is not inferior but rather a valuable strategy for poor responders with insufficient oocyte yield after an initial cycle, thereby broadening opportunities for cryopreservation in time-sensitive oncological settings.

## 1. Introduction

The potential impact of cancer and its treatment, particularly gonadotoxic effects of chemotherapies, on future fertility is a crucial concern in oncological care. As cancer treatment advances and survival rates improve, the need for oncofertility has become increasingly significant. Fertility preservation is particularly relevant for cancer patients, given the rising tumor survival rates in pediatric and adolescent populations and the trend toward delayed childbearing [1,2]. Currently, the main method for fertility preservation is oocyte or embryo cryopreservation; however, the latter option is not universally accessible. In addition, the process is often accelerated to minimize delays in initiating cancer treatment.

International guidelines emphasize discussing the potential consequences on future fertility with patients prior to the initiation of oncological treatments, alongside implementing preventive strategies for fertility preservation [3,4,5]. The primary adverse effect of both chemotherapy and radiotherapy is ovarian toxicity, which can lead to infertility, premature ovarian failure (POF), early menopause and various ovarian endocrine disorders [6].

The ESHRE guidelines recommend oocyte cryopreservation after ovarian stimulation for women undergoing oncological treatment, preferably using a gonadotropin-releasing hormone (GnRH) antagonist protocol, which provides a shorter stimulation period with a favorable safety profile [7]. Recently, alternative protocols have been developed to enhance fertility preservation, yet a standard approach for this specific group of patients has not been established [8].

In emergency ovarian stimulation for fertility preservation, where a shortened stimulation duration is essential before initiating therapy or surgery, the random-start protocol is considered a viable option [9]. This approach is grounded in the follicular wave theory and the continuous recruitment theory, both of which propose that antral follicles are recruited and progress through growth and eventually regression during two or three waves within a menstrual cycle or continuously [3]. Ovarian stimulation for fertility preservation initiated during the luteal phase has shown comparable outcomes to stimulation started in the follicular phase in terms of total gonadotropin dose, stimulation duration, and the number of oocytes retrieved, without needing to wait for the spontaneous onset of menstrual menses [10]. Furthermore, dual stimulation (DuoStim) protocols, involving consecutive stimulations within the same menstrual cycle to increase the number of oocytes retrieved, can be employed [11,12].

Several studies have demonstrated the potential to retrieve oocytes of equivalent quality during both the follicular and the luteal phases, using different stimulation protocols, even following a previous ovarian stimulation in the follicular phase [13]. Therefore, the aim of this study was to compare the effectiveness of controlled ovarian stimulation (COS) protocols performed in different menstrual phases, including DuoStim, in oncologic patients undergoing oocyte preservation, and to identify predictors of mature oocyte yield. The primary outcome was the number of mature (MII) oocytes retrieved. Secondary outcomes included follicular response, oocyte maturity rate, hormonal parameters, and stimulation-related complications.

We hypothesized that COS protocols initiated in different menstrual phases would achieve comparable oocyte yield and quality, and that DuoStim would not be inferior to conventional single-phase stimulations, representing a valuable strategy for patients with time-sensitive oncologic treatments.

## 2. Materials and Methods

### 2.1. Study Design and Population

This retrospective observational single-center study was conducted on oncological patients who were referred to the Fertility Unit of the University Hospital of Pisa between August 2013 and May 2024. All data were obtained from medical records in either paper or electronic formats. The study included patients who underwent oocyte vitrification due to malignant disease requiring treatment and who met the following inclusion criteria according to the Italian public health system: age under 40 years, previous pregnancies or having a child did not preclude participation, a reasonable prognosis of survival (estimated >70% within the subsequent five years), and no contraindications to achieving pregnancy after the completion of oncological treatment.

Exclusion criteria (applied a priori at intake) were age over 40 years, urgent need for cancer treatment within less than two weeks (not allowing ovarian stimulation), advanced disease stage with poor prognosis, contraindications to stimulation (e.g., severe thromboembolic, cardiovascular, pulmonary, hepatic, or renal disease), hematologic or coagulation disorders, prepubertal status, severe comorbidities increasing anesthesia/surgical risk, and poor performance status (ECOG ≥ 3) [14].

**Figure 1:**
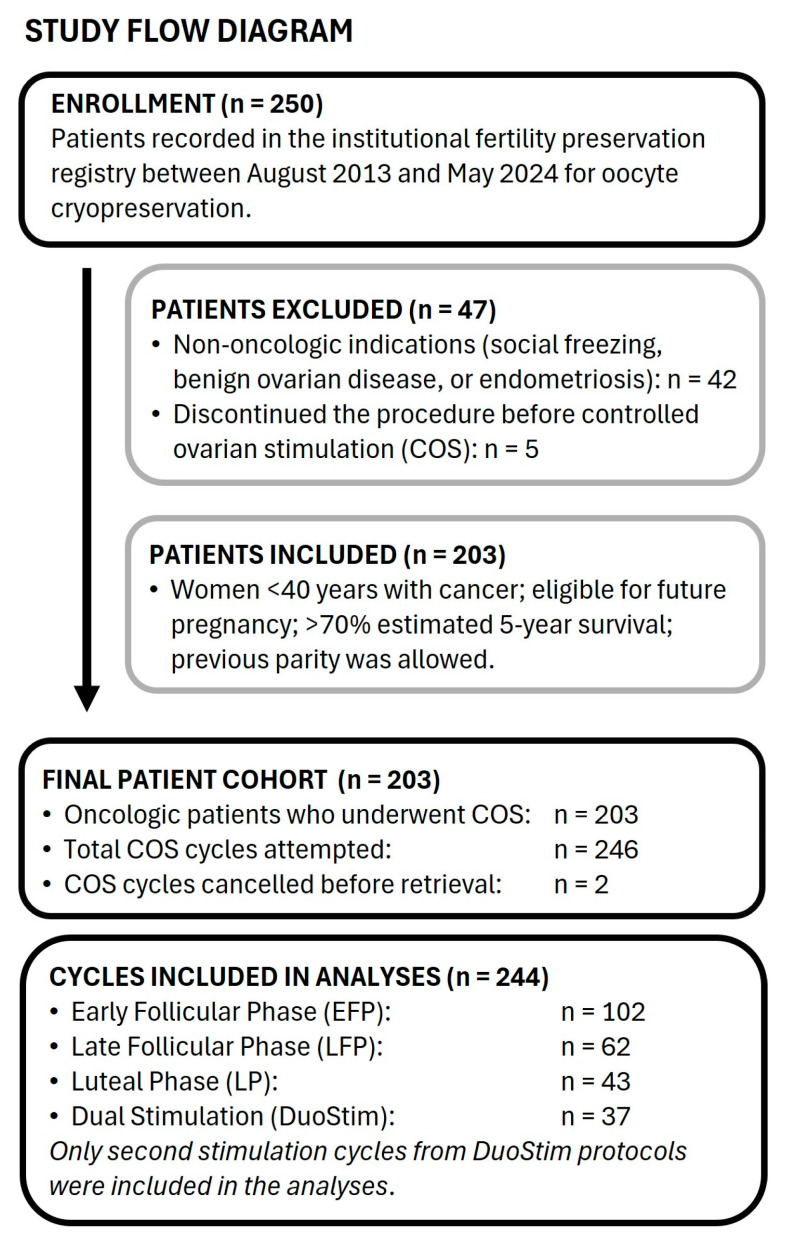
Study flow diagram. The flow chart illustrates the patient inclusion and exclusion criteria throughout the study period, the total number of cycles performed, canceled cycles, and the distribution of cycles included in the analysis, including DuoStim procedures. Only second stimulation cycles from DuoStim protocols were analyzed.

Patients and COS, including DuoStim cases, are summarized in the Study Flow Diagram (Figure 1).

### 2.2. Evaluation and Screening

Each participant underwent an urgent evaluation within a multidisciplinary framework comprising oncologists, gynecologists, and psychologists at the dedicated fertility preservation clinic. Before proceeding with oocyte preservation, comprehensive consultations were conducted to discuss the feasibility of implementing dual stimulation protocols without compromising the commencement of imminent oncological treatments.

Baseline laboratory tests included hormonal assays—follicle-stimulating hormone (FSH, mIU/mL), anti-Müllerian hormone (AMH, ng/mL), estradiol (E2, ng/mL), and progesterone (P4, ng/mL)—as well as infectious disease screening for HIV, hepatitis B surface antigen (HBsAg), total anti-HBc, hepatitis C virus (HCV), syphilis (VDRL/TPHA), rubella (Rubeo test), toxoplasmosis (Toxotest), and cytomegalovirus (anti-CMV).

All patients also underwent a transvaginal ultrasound to assess antral follicle count (AFC) and determine the follicular phase before initiating stimulation.

### 2.3. Stimulation Phase Stratification

Upon presentation to the center, participants were stratified into three distinct cohorts according to their menstrual cycle phase at the onset of stimulation: early follicular phase (EFP), late follicular phase (LFP), and luteal phase (LP). In addition, a four-class classification was applied to define the timing of dual stimulation protocols (DuoStim). Each phase was determined by the gynecologist in order to establish the most appropriate stimulation protocol.

EFP, the initial phase of the ovarian cycle, begins on the first day of menses and is characterized by the visibility of AFC on transvaginal ultrasound and E2 levels below 100 pg/mL. LFP is defined by the presence of at least one dominant follicle measuring between 13 and 17 mm in diameter, as detected by transvaginal ultrasound with concomitant E2 levels ≥ 100 pg/mL and serum P4 levels < 2 ng/mL. The final phase of ovarian cycle, LP, is marked by the presence of the corpus luteum and/or biochemical detection of serum P4 levels exceeding 2 ng/mL.

### 2.4. Controlled Ovarian Stimulation (COS) Protocols

COS protocols were tailored according to the participant’s the menstrual cycle phase. In cases of EFP or LP, stimulation commenced with daily injection of recombinant gonadotropins, such as follitropin alfa, follitropin beta, follitropin delta, corifollitropin alfa, or human menopausal gonadotropin (menotropin, hMG), followed by pituitary suppression with a GnRH antagonist when at least one follicle of 12–14 mm was visualized on transvaginal ultrasound. In cases of LFP, stimulation was initiated with ovulation induction using either a single administration of a GnRH agonist (0.2 mg Triptorelin acetate), a single dose of choriogonadotropin, or 3–5 doses of a GnRH antagonist during the initial days of stimulation. This was then followed by the standard protocol used in the other groups. Final oocyte maturation was triggered 36 hours before oocyte retrieval by administering a GnRH agonist (0.2–0.3 mg).

In women with breast cancer, the addition of letrozole to the standard GnRH antagonist protocol has been shown to effectively mitigate the increase in estrogen levels during ovarian stimulation. This approach has demonstrated no significant differences in the number of mature oocytes retrieved, maturation rate, total gonadotropin dose, or duration of stimulation compared to standard protocols [15]. Consequently, all patients with hormone-sensitive breast cancer were administered 5 mg of letrozole daily throughout the stimulation period. In addition, anticoagulant prophylaxis with 4000 IU of enoxaparin was prescribed from the first day of stimulation until the evening before oocyte retrieval, with the exception of women with a hemorrhagic diathesis, in whom anticoagulation was contraindicated.

### 2.5. DuoStim Protocol

**Figure 2:**
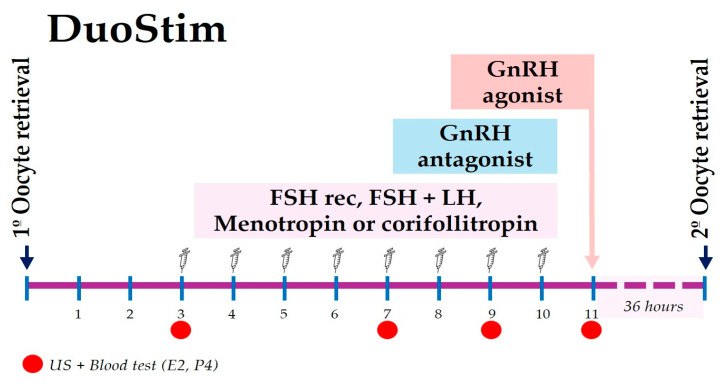
Schematic representation of the DuoStim protocol. The second ovarian stimulation starts on the 3rd–4th day after the first oocyte retrieval, using the same gonadotropin and GnRH antagonist regimen. Final oocyte maturation is triggered with a GnRH agonist 36 h before the second retrieval. Abbreviations: US, ultrasound; E2, estradiol; P4, progesterone; FSH, follicle-stimulating hormone; LH, luteinizing hormone; GnRH, gonadotropin-releasing hormone.

The DuoStim protocol involves two consecutive stimulations aimed at increasing the number of oocytes retrieved. This protocol was offered to women who were able to undergo a second cycle without delaying the initiation of chemotherapy and who had retrieved less than 5 mature oocytes (MII)—or none at all—during the initial stimulation. This protocol begins with the initiation of ovarian stimulation on the third or fourth day following the first oocyte retrieval, utilizing the same antagonist protocol and gonadotropin as employed in the initial COS cycle. Ovarian maturation is induced 36 hours prior to oocyte retrieval by administering a GnRH agonist (0.2–0.3 mg), following the same procedure as in the former COS (Figure 2). All oocytes collected during this second cycle are vitrified separately from those of the first cycle and stored under two distinct identification codes [16].

### 2.6. Outcome Variables

The primary outcome of the study was the number of oocytes that had reached the metaphase II stage following oocyte retrieval, defined as Metaphase II Oocytes (MII). Oocyte maturity was determined by the presence of the first polar body, while oocyte quality was assessed based on morphological features, including cytoplasmic appearance, zona pellucida integrity, and absence of vacuoles or fragmentation [17,18].

Secondary outcomes included the number of follicles > 16 mm at trigger, total oocytes retrieved, oocyte maturity rate, follicular metrics, and ovarian response. Hormonal levels (FSH, AMH at baseline; E2 and P4 at trigger), treatment parameters (gonadotropin dose and duration), and any treatment-related complications were also recorded.

The number of follicles greater than 16 mm in diameter were counted at the day trigger. The total number of oocytes retrieved was documented, and the maturity rate was calculated as the percentage of retrieved oocytes that reached the MII stage out of the total number of oocytes retrieved.

Two follicular metrics were calculated and expressed as percentages: (a) Follicular Output Rate (FORT), defined as the ratio between the number of pre-ovulatory follicles (>16 mm) and the AFC at the beginning of the stimulation cycle [19]; (b) Follicle Oocyte Index (FOI), defined as the ratio of the number of oocytes retrieved and the AFC at the beginning of the stimulation cycle [20]. In addition, a marker of ovarian function, the Ovarian Sensitivity Index (OSI), was calculated as the number of oocytes retrieved divided by the total FSH dose administered, per 1000 IU [21].

At the beginning of the stimulation cycle, AFC and hormonal levels, including FSH and AMH, were determined. E2 and P4 levels were measured on the trigger day. The treatment parameters, such as the numbers of days and the dosage of administered gonadotropins, were reported according to the patient’s treatment log.

Throughout the treatment cycle, all complications were closely monitored and recorded by the attending clinicians according to standard institutional protocols. Adverse events included Ovarian Hyperstimulation Syndrome (OHSS), infections, bleeding, or any other clinically relevant events and were systematically documented in the patients’ medical records and subsequently reviewed for the purpose of this study.

### 2.7. Ethical Approval

All data presented in this study were collected as part of a service evaluation of the fertility preservation program, in accordance with established Italian protocols and best practices for Patient-Reported Outcome Measures (PROMs) and Patient-Reported Experience Measures (PREMs) [22,23]. As the data originated from routine clinical evaluations performed under institutional policies, formal approval from an ethics committee was not required. Nonetheless, strict measures were implemented to ensure patient confidentiality and compliance with data protection regulations. Informed consent was obtained from all participants, who were clearly informed about the voluntary nature of their participation and the intended use of their data. Before analysis, all information was anonymized, and data handling procedures strictly followed the European General Data Protection Regulation (GDPR—EU 2016/679) [24]. The study was conducted in accordance with the ethical principles outlined in the Declaration of Helsinki [25].

### 2.8. Statistical Analysis

#### 2.8.1. Descriptive and Comparative Analyses

The data was analyzed using GraphPad Prism 10 (GraphPad Software, Boston, MA, USA). Categorical variables were summarized as absolute frequencies (number, n) and relative frequency (percentage, %). Continuous variables were expressed as mean ± standard deviation (SD) when normally distributed, and as median with interquartile range (IQR, 25th–75th percentile) when the distribution deviated from normality.

Normality was assessed using both the Shapiro–Wilk test for smaller sample sizes (*n* < 50) and graphical methods (Q–Q plots, histograms, and density plots) for larger groups. For non-normally distributed variables, comparisons across the four groups were performed using the two-tailed Kruskal–Wallis test, followed by Dunn’s multiple comparisons test. To account for multiple testing, *p*-values were adjusted using the Benjamini–Hochberg false discovery rate (FDR) procedure. In the tables, adjusted results are reported as adjusted *p*-values (FDR).

#### 2.8.2. Multivariable Regression Analysis

To explore potential sources of bias in predicting the number of mature (MII) oocytes retrieved, both multiple linear regression and Poisson regression models were applied, with the latter chosen to account for the count nature of the outcome. Candidate predictors included age, year of cryopreservation, basal FSH, AMH, AFC, E2 at trigger, and the follicular phase at the start of stimulation (EFP, LFP, LP, and DuoStim). These variables were selected based on their relevance in the literature and their potential to introduce temporal or biological variability. Model fit was assessed using likelihood ratio tests, and multicollinearity was evaluated using variance inflation factors (VIF).

For all comparisons, adjusted *p*-values < 0.05 were considered statistically significant.

## 3. Results

### 3.1. Baseline Characteristics and Oncological Distribution

A total of 250 patients were registered during the study period. Among them, 42 had non-oncologic indications (social freezing or benign ovarian disease, including endometriosis), and 5 discontinued the procedure before COS. The final cohort comprised 203 oncologic patients who underwent COS for oocyte cryopreservation and were included in the present analysis, providing a total of 246 initial cycles. However, oocyte retrieval was not performed in two cases due to personal and clinical reasons, resulting in a total of 244 cycles from 203 patients for analysis (Figure 1).

Table 1 and Table 2 provide a comprehensive overview of the baseline characteristics and the distribution of oncological diagnoses among the patients. The median age of the cohort was 31.0 years (IQR: 27.0–35.0). Breast cancer was the most prevalent diagnosis, accounting for 96 patients (47.3%) and 118 cycles (48.4%). Lymphomas were diagnosed in 65 patients (32.0%) contributing to 70 cycles (28.7%). Gynecological cancers (including ovarian, endometrial, and cervical) were observed in 14 patients (6.9%) with 21 cycles (8.6%). Colon cancer was present in 7 patients (3.4%) with 7 cycles (2.9%), while other types of cancer were identified in 13 patients (6.4%) corresponding to 15 cycles (6.1%). Additionally, 8 patients (3.9%) undergoing chemotherapy for other pathologies contributed to 13 cycles (5.3%) (Table 1). The median duration of gonadotropin treatment was 9 days (IQR 8–10). Compliance-related complications were reported in 10 cases, representing 4.12% of the total cycles (Table 2).

### 3.2. Comparison of Stimulation Protocols Cohorts Across Oncological Patients

The stimulation cycles were categorized into four distinct cohorts based on the follicular phase at the initiation of stimulation. The distribution of cycles across these cohorts was as follows: EFP with 102 cycles, LFP with 62 cycles, LP with 43 cycles, and DuoStim with 37 cycles.

No significant differences were observed in age, FSH or AMH levels, or complication rates among the four cohorts. However, the DuoStim group demonstrated a significantly lower number of AFC compared to the EFP, LFP and LP groups (all adjusted *p* < 0.01, FDR). At the time of induction, P4 levels did not differ significantly among the groups; however, E2 levels were significantly lower in the DuoStim group compared to the EFP, LFP and LP groups (adjusted *p* < 0.01, <0.01, and <0.05, respectively, FDR). In addition, the LP group required a significantly higher number of days of gonadotropin administration (all adjusted *p* < 0.001, FDR) and received a higher total dose of gonadotropins compared to the other groups (all adjusted *p* < 0.01, FDR) (Table 2).

The median number of follicles greater than 16 mm, as well as the FORT, did not differ significantly across the groups. The DuoStim group showed a significantly lower median number of oocytes retrieved and OSI (all adjusted *p* < 0.001, FDR) compared to the other groups. Similarly, the median number of MII oocytes was reduced in DuoStim (3.0, IQR 2.0–5.0) compared to the EFP (5.0, IQR 3.0–8.0), LFP (6.0, IQR 3.0–9.0), and LP (6.0, IQR 3.0–9.0) groups (adjusted *p* < 0.05, <0.01, and <0.01, respectively, FDR). It should be noted that the table reports only the oocytes collected and analyzed from the second stimulation; when oocytes from both the first and second stimulations are combined (5.0, IQR 2.5–8.0), no significant differences are observed among the groups. The follicle output FOI, and oocyte maturity rate did not show a significant difference among the cohorts (Table 2).

### 3.3. Outcomes in the DuoStim Subgroup

Among the 37 women who underwent DuoStim, 24 initiated stimulation with an EFP protocol, 8 with an LPF, and 5 with an LP protocol. In this cohort, nine women (24.3%) did not obtain mature oocytes (MII) during the first stimulation, including one in whom oocyte retrieval could not be performed. All nine subsequently underwent DuoStim, achieving a median of 3 oocytes retrieved (IQR 1.2–7.8) and 3 mature oocytes (IQR 1.2–5.7), with a maturity rate of 100% (IQR 69.9–100). Only one patient did not obtain oocytes in either stimulation.

In the remaining 28 cases, 6 (16.2%) patients did not yield oocytes after the second stimulation. One of these underwent a third stimulation (TriStim) due to a combination of an insufficient oocyte yield and time availability.

Overall, the cumulative DuoStim nearly doubled the number of mature oocytes compared with the first stimulation (mean ± SD: 5.7 ± 4.3 vs. 2.6 ± 3.3 MII oocytes).

DuoStim implementation increased progressively in our center (1 patient in 2017; 4 in 2018; 7 in 2019; 6 in 2020). Since 2021, it has been systematically offered when oncologic timing permits, particularly when fewer than 10 mature oocytes are obtained after the first stimulation. Cases in which DuoStim was not performed after 2021 were due exclusively to insufficient time before cancer treatment. The stimulation protocol used in our center is illustrated in Figure 2.

### 3.4. Outcomes in Breast Cancer Patients

Breast cancer was the most prevalent indication for fertility preservation in our study, prompting a focused analysis of this group to gain a deeper insight into their characteristics. Table 3 presents the baseline characteristics of breast cancer patients across the cohorts as follows: EFP with 45 cycles, LFP with 28 cycles, LP with 22 cycles, and DuoStim with 23 cycles. Consistent with the overall cohort findings, no significant differences were observed in age, FSH levels, AMH levels or complication rates among the groups. However, the DuoStim group showed a significantly lower number of AFC compared to the EFP, LFP and LP groups (adjusted *p* < 0.05, <0.05, and <0.01, respectively, FDR).

Regarding oocyte preservation, the DuoStim group exhibited a lower median number of oocytes retrieved compared to the LP group (adjusted *p* < 0.05, FDR). The median number of MII oocytes collected during the second stimulation was 3.0 (IQR 1.0–6.0) for DuoStim, 5.0 (IQR 3–9) for EFP, 4.0 (IQR 2.0–7.2) for LFP, and 6.0 (IQR 3.0–8.2) for LP; these differences were not statistically significant. It should be noted that the table reports only the oocytes collected and analyzed from the second stimulation; when oocytes from both the first and second stimulations are combined, the median in the DuoStim group increases to 5.0 (IQR 3–9), yet no significant differences are observed among the groups. Despite these variations in absolute numbers, the median number of follicles greater than 16 mm, FORT, FOI, OSI, and oocyte maturity rate did not differ significantly among the cohorts.

### 3.5. Multivariable Regression Results

To explore potential sources of bias in predicting the number of MII oocytes retrieved, a multivariable regression analysis was performed. Both linear and Poisson models were tested, with consistent results; therefore, only the Poisson regression is reported. Candidate predictors included age, year of cryopreservation, basal FSH, AMH, AFC, E2 at trigger. In this model only AMH (β = 0.085, 95% CI 0.046–0.124, *p* < 0.001) and AFC (β = 0.060, 95% CI 0.041–0.078, *p* < 0.001) were independently associated with the number of mature oocytes retrieved (Table 4). Neither age, basal FSH, E2 at trigger, the year of cryopreservation showed significant associations. The model showed no evidence of multicollinearity (all VIF < 2) and explained approximately 43% of the variance in MII oocyte count (pseudo R^2^ = 0.43). These findings confirm that ovarian reserve markers, specifically AMH and AFC, are the principal determinants of oocyte yield, while demographic, temporal, and hormonal variables exerted no significant influence.

## 4. Discussion

Controlled ovarian stimulation (COS) is a well-established strategy for fertility preservation in oncological patients and can be performed without delaying anticancer treatments. Our findings confirm that DuoStim achieves a cumulative number of mature oocytes comparable to conventional random-start protocols without compromising oncologic treatment timelines. This observation is consistent with previous reports demonstrating that consecutive stimulations within the same menstrual cycle do not compromise oocyte competence, thereby supporting the follicular wave theory and the rationale for flexible stimulation approaches in urgent oncofertility scenarios [26,27,28].

From a clinical perspective, DuoStim should not be considered an inferior approach but rather a valuable rescue strategy for poor responders, especially when the first stimulation yields an insufficient number of mature oocytes. Performing an immediate second stimulation within the same cycle maximizes oocyte yield while maintaining oncologic safety—an advantage previously demonstrated in both IVF and oncofertility contexts [16,28,29].

In one case, a triple stimulation (TriStim) was performed to maximize oocyte retrieval without compromising the timing of oncological treatment. Across three stimulations in 33 days, the patient obtained a total of six mature oocytes (one in the first EFP cycle, none in the second phase of DuoStim, and five following the third stimulation), experiencing only mild OHSS with no delay to the initiation of cancer treatment. Although the use of TriStim is not widely supported by extensive data, its successful application in this case highlights its potential as an emergency strategy for oocyte preservation. Particularly before radical procedures like bilateral oophorectomy, which eliminate the possibility of using one’s own oocytes after treatment [30]. Nevertheless, TriStim should be considered exceptional and tailored to the individual, pending further data.

Although the molecular mechanisms behind DuoStim are not yet fully understood, current evidence supports the concept that the ovary undergoes multiple follicular waves within a single cycle [3,31]. Clinically, this approach offers an opportunity to increase the total number of oocytes obtained within a limited time frame, which is particularly valuable in urgent oncofertility settings.

Breast cancer, the most common indication for oncofertility, represented the largest subset of patients in the study. In our cohort, despite lower baseline AFC, DuoStim achieved comparable maturity rates and cumulative yields across all stimulation phases. Although the median number of retrieved oocytes was slightly lower (3.0, IQR 1.0–6.0) than in single-phase protocols (4.0–6.0), the maturity rate remained similar (83.3% vs. 73.9–85.7%), and both parameters were found to be not statically significant. These results support DuoStim as an effective, time-sensitive option for urgent fertility preservation in women with limited ovarian reserve [3,10,32].

The observed outcomes consistent with those of earlier studies, including those by Moffat et al. (2014) and Tsampras et al. (2017), which highlight the ability to retrieve a comparable number of oocytes across different phases, thus supporting the utility of flexible stimulation protocols in urgent oncofertility settings [28,33]. The DuoStim protocol aligns with existing literature, which indicates lower E2 levels, a reduction in the number of oocytes, and a decrease in the number of mature oocytes in comparison to the first COS [34]. This suggests that the follicular response diminishes with successive stimulations, although oocyte quality, as reflected in the maturity rates, remains intact [35].

This study observed a notably higher gonadotropin requirement and a longer stimulation duration in the luteal phase (LP) compared to EFP, and LFP. These findings are in line with those of some recent studies that reported significantly increased gonadotropin use in LP cycles, likely reflecting reduced follicular sensitivity during this phase, particularly in patients with low ovarian reserve [36].

It is important to acknowledge that patients undergoing DuoStim in our cohort presented lower baseline AFC compared with those undergoing single stimulation approaches, consistent with the clinical selection of poorer responders for this protocol. However, AMH levels were comparable between groups, suggesting that the observed differences in oocyte yield are likely influenced by antral follicle availability rather than endocrine markers of ovarian reserve. Therefore, the performance of DuoStim in this setting should be interpreted within the context of inherently more challenging ovarian biology.

A comparison of these results with existing literature reveals both consistencies and novel insights. In this study, for instance, follicular metrics such as FORT, FOI, and OSI were utilized as parameters to assess the follicular response to stimulation. While the various COS-1 stimulation phases did not yield substantial variance in the parameters, a modest discrepancy was discerned between DuoStim and the alternative COS-1 stimulation protocols. However, within the breast cancer cohort, this distinction was not sustained, as all parameters demonstrated no significant differences among the various protocols, including DuoStim.

The present study found that the DuoStim protocol did not result in significant early or late complications. In a related context, the challenge of immature oocytes, a condition described in the literature but with unclear etiology, prompted us to modify our ovulation induction protocol following episodes of unsuccessful oocyte retrieval despite optimal ultrasound and hormonal dosage stimulation [37]. Consequently, we doubled the dosage of the GnRH agonist for oocyte maturation, increasing Triptorelin from 0.2 mg to 0.3 mg, administered 36 hours before oocyte retrieval. Additionally, in patients with a low risk of OHSS, we occasionally supplemented with 1500 IU of human chorionic gonadotropin (HCG) 12 h before retrieval. Although studies have shown that double induction with GnRH agonists and HCG can lead to higher oocyte maturation and fertilization rates, this effect has not yet been conclusively demonstrated in oncological patients [38].

A recent SWOT (strengths, weaknesses, opportunities and threats) analysis of GnRH agonist uses for ovarian stimulation found that it induces rapid luteolysis, creating a favorable hormonal environment for subsequent stimulation cycles [39]. This technique does not compromise the recovery of new follicular waves, allowing for consecutive stimulations. Additionally, Vaiarelli et al. demonstrated that oocyte competence is comparable between Norethisterone Acetate Progestin-Primed Ovarian Stimulation (NETA-PPOS) and the conventional GnRH antagonist protocol [40]. Future research should investigate the efficacy of NETA-PPOS protocols in oncological patients, as they may reduce the number of injections, decrease dropout rates, and reduce the psychophysical and economic burden on patients [39].

The principal strength of this study is its large sample size and 10-year recruitment period, which enhance the validity of the results. The comprehensive evaluation of multiple stimulation protocols in oncologic patients, with a particular focus on breast cancer patients, allowed for the identification of trends based on menstrual cycle phases. The use of standardized COS protocols and meticulous outcome documentation, encompassing oocyte counts, follicular metrics, and hormone levels, serves to reinforce the reliability of the findings.

### Limitations and Future Perspectives

This study is limited by its retrospective, single-center design and the absence of long-term reproductive outcomes. Although COS protocols were standardized, the choice of protocol may have been influenced by clinical urgency and ovarian reserve. Despite a long recruitment period, only 10 of 203 patients (4.9%) have returned to use their cryopreserved oocytes to date, which limits the evaluation of clinical outcomes such as pregnancy and live birth rates. Furthermore, the results may not be generalizable to centers using different stimulation approaches or laboratory conditions.

Future prospective multicenter studies with long-term follow-up are required to assess reproductive and live-birth outcomes in oncology patients. Such studies will facilitate the improvement of stimulation protocols, including the adjustment of GnRH-agonist dose or the optimization of NETA-PPOS trigger settings.

## 5. Conclusions

The present study demonstrates the efficacy of COS protocols for fertility preservation in oncological patients regardless of menstrual cycle phase and shows that they do not delay cancer treatment. In our experience, flexible stimulation strategies, particularly DuoStim, are valuable in urgent oncofertility settings. These allow patients with low ovarian reserve or limited time before oncologic therapy to maximize oocyte yield within a single cycle.

In circumstances where time is available, conventional single-phase stimulation remains appropriate; however, DuoStim represents a practical and effective rescue option in patients facing imminent gonadotoxic treatment. In extremely time-constrained scenarios, a third stimulation cycle (TriStim) may be considered on an individualized basis, although the current evidence remains anecdotal.

## Figures and Tables

**Table 1:** Baseline patient characteristics according to diagnosis. Values are reported as absolute numbers, with percentages in parentheses, calculated with respect to the total number of cycles and to the total number of patients, respectively. Gynecological cancer (Malignant Ovarian, Endometrial, and Cervical Cancer).

Diagnosis	Total Number of Cycles (%)	N patients (%)
Breast Cancer	118 (48.4)	96 (47.3)
Lymphoma	70 (28.7)	65 (32.0)
Gynecological cancer	21 (8.6)	14 (6.9)
Colon Cancer	7 (2.9)	7 (3.4)
Other Cancers	15 (6.1)	13 (6.4)
Chemotherapy for other Pathologies	13 (5.3)	8 (3.9)

**Table 2:** Outcome variables related to controlled ovarian stimulation in different phases of the menstrual cycle. Categorical variables are expressed as absolute frequency (n) and relative frequency (%), while continuous variables are reported as median and interquartile range (IQR, 25th–75th percentile). Categorical variables were analyzed with Fisher’s exact test. Comparisons among the four groups were performed using the two-tailed Kruskal–Wallis test followed by Dunn’s post hoc test. To control all multiple comparisons, *p*-values were adjusted using the Benjamini–Hochberg false discovery rate (FDR) procedure, and results are reported as adjusted *p*-values (FDR). (* *p* < 0.05, ** *p* < 0.01, *** *p* < 0.001, *ns*: non-significant). EFP (Early Follicular Phase); LFP (Late Follicular Phase); LP (Luteal Phase); FSH (Follicle-Stimulating Hormone); AMH (Anti-Müllerian Hormone); AFC (Antral Follicle Count); E2 (Estradiol); P4 (Progesterone); FOI (Follicular Output Rate); FORT (Follicular Output Rate); OSI (Ovarian Sensitivity Index); MII (Metaphase II Oocytes).

	Overall Cohort	EFP (a)	LPF (b)	LP (c)	DuoStim (d)	*p*-Value (FDR)
Total Number of Cycles	244	102	62	43	37	
Age (years)	31.0 (27.0–35.0)	31.0 (26.0–35.0)	32.0 (24.0–35.3)	30.0 (26.0–34.0)	32.0 (29.0–36.0)	*ns*
FSH (mUI/mL)	6.80 (4.3–8.8)	6.7 (4.2–8.7)	6.3 (3.7–8.1)	7.5 (5.9–11.7)	7.6 (4.2–8.8)	*ns*
AMH (pg/mL)	2.2 (1.0–3.6)	2.0 (0.9–3.6)	2.7 (1.8–3.8)	2.3 (1.0–4.3)	1.7 (0.9–3.1)	*ns*
AFC	10 (7.0–15.0)	10 (7.0–17.0)	10.5 (8.0–15.0)	11 (9.0–18.0)	8 (5.0–10.0)	*d* vs. a **, b **, c **
Gonadotropin (days)	9 (8–10)	8.0 (8.0–9.0)	9.0 (9.0–9.0)	10.0 (10.0–13.0)	8.0 (8.0–9.0)	*c* vs. a ***, b ***, d *** a vs. b *
Total Gonadotropin (IU)	2700 (2400–3000)	2400 (2250–2700)	2700 (2594–2700)	3000 (2700–3300)	2700 (2400–3000)	*a* vs. b **, d * c vs. a ***, b **, d **
E2 trigger (pg/mL)	572 (229–1176)	619 (295–1188)	693 (298–1295)	577 (216–1099)	232 (115–652)	*d* vs. a **, b **, c *
P4 trigger (pg/mL)	1.34 (0.88–2.12)	1.26 (0.58–1.97)	1.53 (0.90–2.49)	1.35 (0.98–2.39)	1.40 (1.03–2.08)	*ns*
FOI	0.60 (0.38–0.87)	0.58 (0.39–0.90)	0.63 (0.38–0.82)	0.63 (0.45–0.89)	0.43 (0.17–0.78)	*ns*
FORT	0.40 (0.27–0.59)	0.40 (0.29–0.60)	0.40 (0.25–0.51)	0.35 (0.21–0.56)	0.42 (0.32–0.80)	*ns*
OSI	2.45 (1.30–3.88)	2.50 (1.40–4.70)	2.50 (1.50–4.40)	2.70 (1.50–3.90)	1.40 (0.50–2.35)	*d* vs. a ***, b ***, c ***
Follicles ≥ 16 mm	4.0 (2.0–6.0)	4.0 (2.0–7.0)	4.0 (2.0–5.25)	4.0 (2.0–6.0)	4.0 (2.0–5.0)	*ns*
Oocytes Retrieved	6.0 (3.0–10.0)	6.5 (3.8–10.0)	7.0 (4.0–11.2)	8.0 (4.0–12.0)	3.0 (1.5–6.0)	*d* vs. a ***, b ***, c ***
MII Oocytes	5.0 (3.0–8.0)	5.0 (3.0–8.0)	6.0 (3.0–9.0)	6.0 (3.0–9.0)	3.0 (2.0–5.0)	*d* vs. a *, b **, c **
Maturity Rate %	83.4 (60.3–100.0)	87.5 (60.0–100.0)	80.0 (58.3–100.0)	75.0 (60.8–93.8)	92.9 (65.6–100.0)	*ns*
Compliance	10 (4.12%)	4 (3.9%)	4 (6.4%)	1 (2.3%)	1 (2.7%)	*ns*

**Table 3:** Outcome variables in the subgroup of breast cancer related to controlled ovarian stimulation in different phases of the menstrual cycle. Categorical variables are expressed as absolute frequency (n) and relative frequency (%), while continuous variables are reported as median and interquartile range (IQR, 25th–75th percentile). Categorical variables were analyzed with Fisher’s exact test. Comparisons among the four groups were performed using the two-tailed Kruskal–Wallis test followed by Dunn’s post hoc test. To control all multiple comparisons, *p*-values were adjusted using the Benjamini–Hochberg false discovery rate (FDR) procedure, and results are reported as adjusted *p*-values (FDR) (* *p* < 0.05, ** *p* < 0.01, *** *p* < 0.001, *ns*: non-significant). Abbreviations: EFP (Early Follicular Phase); LFP (Late Follicular Phase); LP (Luteal Phase); FSH (Follicle-Stimulating Hormone); AMH (Anti-Müllerian Hormone); AFC (Antral Follicle Count); E2 (Estradiol); P4 (Progesterone); FOI (Follicular Output Rate); FORT (Follicular Output Rate); OSI (Ovarian Sensitivity Index); MII (Metaphase II Oocytes).

	EFP (a)	LPF (b)	LP (c)	DuoStim (d)	*p*-Value (FDR)
Total Number of Cycles	45	28	22	23	
Age (years)	34.5 (31.0–36.0)	35.0 (32.0–37.8)	34.0 (31.5–35.3)	35 (31.0–37.0)	*ns*
FSH (mUI/mL)	7.5 (4.1–9.65)	7.4 (4.9–10.5)	7.1 (4.13–10.6)	8.2 (4.2–9.5)	*ns*
AMH (pg/mL)	2.1 (0.9–4.5)	2.4 (1.6–35)	2.4 (0.9–4.2)	1.9 (1.0–4.0)	*ns*
AFC	10 (7.0–15.0)	12.0 (8.0–15.0)	11 (9.0–17.5)	8 (5.0–10.0)	*d* vs. a *, b *, c **
Gonadotropin (days)	8.0 (8.0–9.0)	9.0 (9.0–9.0)	10.0 (9.0–11.0)	8.0 (8.0–9.0)	*c* vs. a ***, b ***, c ***
Total Gonadotropin (IU)	2400 (2400–2700)	2700 (2700–2700)	3000 (2981–3350)	2700 (2400–3000)	*c* vs. a ***, b **, d *** *a* vs. b *
E2 trigger (pg/mL)	326 (209–615)	318 (195–530)	272 (156–690)	145 (77–367)	*d* vs. a *
P4 trigger (pg/mL)	1.38 (0.92–2.22)	1.79 (1.33–3.35)	1.61 (1.06–2.38)	1.5 (1.07–2.33)	*ns*
FOI	0.62 (0.40–0.90)	0.53 (0.32–0.80)	0.63 (0.50–0.89)	0.60 (0.40–1.00)	*ns*
FORT	0.33 (0.20–0.50)	0.36 (0.22–0.50)	0.35 (0.26–0.46)	0.50 (0.33–0.90)	*ns*
OSI	2.80 (1.40–4.05)	1.95 (1.30–3.05)	2.70 (1.45–3.73)	1.50 (0.80–2.50)	*ns*
Follicles ≥ 16 mm	3.0 (2.0–5.0)	4.5 (2.0–5.75)	4.0 (2.0–6.0)	3.0 (2.0–5.0)	*ns*
Oocytes Retrieved	6.0 (3.0–10.0)	5.0 (4.0–8.5)	8.0 (4.0–11.2)	4.0 (2.0–6.0)	*d* vs. c *
MII Oocytes	5.0 (2.0–8.0)	4.0 (2.0–7.2)	6.0 (3.0–8.2)	3.0 (1.0–6.0)	*ns*
Maturity Rate %	81.5 (53.0–100.0)	85.7 (55.3–100.0)	73.9 (59.6–92.5)	83.3 (50.0–100.0)	*ns*
Compliance	2 (4.4%)	2 (7.14%)	0 (0.0%)	1 (4.4%)	*ns*

**Table 4:** Multivariable regression analysis of predictors of MII oocyte retrieved. Results of the multivariable Poisson regression model assessing potential predictors of MII oocyte count. Model diagnostics indicated no relevant multicollinearity (all VIF < 2) with an overall model fit of pseudo R^2^ = 0.43. Abbreviations: MII (Metaphase II Oocytes), FSH (Follicle-Stimulating Hormone); AMH (Anti-Müllerian Hormone); AFC (Antral Follicle Count); E2 (Estradiol).

Predictor MII Retrieved	Estimate β	95% CI	*p*-Value
Year of cryopreservation	−0.023	−0.061–0.015	0.351
Age (years)	−0.003	−0.019–0.013	0.673
FSH (mIU/mL)	−0.014	−0.036–0.007	0.233
AMH (ng/mL)	0.085	0.046–0.124	<0.001
AFC	0.060	0.041–0.078	<0.001
E2 at trigger (pg/mL)	−2.6 × 10^−5^	−9.3 × 10^−9^–3.8 × 10^−5^	0.424

## Data Availability

The data presented in this study are available upon request from the corresponding author due to ethical reasons and patient confidentiality. Access is restricted in accordance with institutional policies and data protection regulations.

## Abbreviations

The following abbreviations are used in this manuscript:

COS	Controlled Ovarian Stimulation
DuoStim	Dual Stimulation
BC	Breast Cancer
EFP	Early Follicular Phase
LFP	Late Follicular Phase
LP	Luteal Phase
POF	Premature Ovarian Failure
AFC	Antral Follicle Count
AMH	Anti-Müllerian Hormone
MII	Metaphase II Oocytes
FORT	Follicular Output Rate
FOI	Follicle Oocyte Index
OSI	Ovarian Sensitivity Index
